# Reducing inequities in maternal and child health in rural Guatemala through the CBIO+ Approach of Curamericas: 4. Nutrition-related activities and changes in childhood stunting, wasting, and underweight

**DOI:** 10.1186/s12939-022-01756-8

**Published:** 2023-02-28

**Authors:** Henry B. Perry, Ira Stollak, Ramiro Llanque, Stanley Blanco, Elizabeth Jordan-Bell, Alexis Shindhelm, Carey C. Westgate, Andrew Herrera, Mario Valdez

**Affiliations:** 1 Health Systems Program, Department of International Health, Johns Hopkins Bloomberg School of Public Health, Baltimore, Maryland USA; 2 Curamericas Global, Raleigh, North Carolina USA; 3 Consejo de Salud Rural Andino/Curamericas, La Paz, Bolivia; 4 Research Triangle Institute, Research Triangle, Raleigh, North Carolina USA; 5 Department of Neurology, Duke University Medical Center, Durham, North Carolina USA; 6 Community Health Impact Coalition, New York, New York USA; 7 Curamericas Guatemala, Calhuitz, San Sebastián Coatán, Huehuetenango, Guatemala

**Keywords:** Maternal health, Child health, Child nutrition, Community health, Primary health care, Community-based primary health care, Implementation research, Census-Based, Impact-Oriented Approach, Care Groups, Community Birthing Centers, Guatemala, Equity, Curamericas Global, Curamericas/Guatemala

## Abstract

**Background:**

This is the fourth paper in our supplement on improving the health and well-being ﻿of rural indigenous Maya mothers and children in the Western Highlands of Guatemala, where the prevalence of stunting is the highest in Latin America and among the highest in the world. Reducing childhood undernutrition was one of the objectives of the Maternal and Child Health Project, 2011–2015, implemented by Curamericas/Guatemala. The implementation research portion of the Project attempted to determine if there were greater improvements in childhood nutritional status in the Project Area than in comparison areas and whether or not a dose–response effect was present in terms of a greater improvement in the Project Area with a longer duration of interventions.

**Methods:**

The Project provided nutrition-related messages to mothers of young children, cooking sessions using locally available nutritious foods, a lipid-based nutrient supplement (Nutributter®) for a short period of time (4 months)﻿, anti-helminthic medication, and repeated growth monitoring and nutrition counseling. Measures of height and weight for calculating the prevalence of underweight, stunting, and wasting in under-2 children were analyzed and compared with the anthropometric data for children in the rural areas of the Northwestern Region and in the Western Highlands of Guatemala.

**Results:**

The prevalence of stunting declined in Area A from 74.5% in September 2012 to 39.5% in June 2015. Area A comprised approximately one-half of the Project Area and was the geographic area with the greatest intensity and duration of nutrition-related Project interventions. Minimal improvements in stunting were observed in the Northwestern Region, which served as a comparison area. Improvements in multiple output and outcome indicators associated with nutritional status were also observed in Areas A and B: infant and young child feeding practices, routine growth monitoring and counseling, and household practices for the prevention and treatment of diarrhea.

**Conclusion:**

The Project Area in which Curamericas/Guatemala implemented the CBIO+ Approach experienced a reduction in the prevalence of stunting and other measures of undernutrition in under-2 children. Given the burden of undernutrition in Guatemala and other parts of the world, this approach merits broader application and further evaluation.

## Background

This is the fourth paper in our supplement assessing the effectiveness of the CBIO+ Approach in improving the health of the Indigenous Maya women and children in the rural mountainous highlands of the Department of Huehuetenango. The term CBIO+ refers to the Expanded Census-Based, Impact-Oriented Approach that includes Care Groups and Community Birthing Centers called *Casas Maternas Rurales*. The first paper [1] introduced the supplement and described the CBIO+ methodology as well as its applications in the Maternal and Child Health Project (hereafter referred to as the Project). It also provides detailed information on the inequities suffered by this population group and notes the high prevalence of childhood stunting experienced by the children in this population group relative to non-indigenous children in Guatemala and children in other parts of the world. The second paper [2] described the study site, the research design, and the methods used for data collection. The third paper [3] described the changes in the population coverage of key interventions during Project implementation. This article describes the Project’s nutrition-related activities and documents the changes in childhood nutritional status that were observed. The other papers in the supplement focus on the assessment of mortality [4], assessment of the quality of care provided at Community Birthing Centers [5], assessment of women’s empowerment [6, 7], assessment of key stakeholders’ perspectives on strengthening the CBIO+ approach [8], and cost-effectiveness and broader policy implications [9].

Linear growth is now well-recognized as an excellent indicator of child health and well-being at the population level [10, 11]. Linear growth faltering (also referred to as stunting), when present, is often particularly pronounced during the first two years of life [12]. Its causes include poor maternal health and nutrition, persistent suboptimal feeding practices, and recurrent infections, with poverty being an underlying contributor to all of these [10]. Undernutrition compromises immune system function and is a contributing (or underlying) cause of approximately one-half of under-5 mortality in low- and middle-income countries [10]. Furthermore, stunting is associated with other consequences that are long-term: shorter adult height, reduced school achievement, and reduced economic outputs, all of which contribute to stunting in the next generation [13]. As a consequence, stunting is widely considered to be the best overall indicator of suboptimal child welfare and an accurate marker of poor child development and social inequalities at the population level [14].

A significant challenge to improving the health of children younger than 5 years of age (referred to hereafter as under-5 children) in the rural indigenous Maya population of Guatemala has been the very high prevalence of stunting. Guatemala has the sixth highest prevalence of stunting in the world and the highest in Latin America [15]. A recent review of anthropometric surveys from 13 countries in Latin America indicates that under-5 children in Guatemala have by far the highest prevalence of stunting among these countries (46.7% compared to the next highest, which is 24.6% in Ecuador) [16]. Among the indigenous ethnic minorities of these 13 countries, the prevalence of stunting among indigenous children of Guatemala is 61.4% while Ecuador has the next highest level – 41.8%. According to the most recent Guatemala Demographic and Health Survey (2014/15), in the Department of Huehuetenango, where the Project is located and which has an overwhelmingly rural Maya population, 67.7% of under-5 children were stunted [17]. A 2013 survey conducted by the Western Highlands Integrated Project (WHIP) in 20 rural municipalities of the predominantly Maya Western Highlands, which includes the Department of Huehuetenango as well as other departments in western Guatemala, revealed similar findings: 67.4% of under-5 children were stunted [18]. Contributing to this situation have been household food insecurity;﻿ large family size; history of diarrhea; lack of knowledge about proper nutrition for infants and children;﻿ endemic poverty;﻿ an over-dependence on maize (corn);﻿ a cultural belief that maize alone is sufficient nutrition;﻿ poor maternal nutrition during pregnancy;﻿ and mothers’ lack of money, time, and control over household finances and decisions [19–22].

### Project nutrition-related activities

The Project utilized the CBIO+ Approach – a joint set of approaches combining the Census-Based, Impact-Oriented Approach with the Care Group Approach and the Community Birthing Center Approach – to engage communities in a joint effort to improve the health of mothers and children. Because of the high prevalence of stunting in the Project Area, 30% of the Project’s level of effort was devoted to addressing the high prevalence of stunting in under-2 children through (1) community-based growth monitoring and counseling on appropriate child feeding practices, (2) peer-to-peer nutrition education through Care Groups, and (3) special support for children with growth faltering. These interventions were implemented in the 91 communities of Project Area A from March 2012 through May 2015 and in the 89 communities of Project Area B from October 2013 through May 2015.

### Growth monitoring, counseling, and other support

A census-based growth-promotion approach reaching all children in their homes was utilized. Level-1 and Level-2 Care Group Promoters (described further in Paper 1 [1]) were trained in anthropometry. The Promoters periodically measured the height^1^ and weight of under-2 children. Utilizing community registers of under-2 children and community maps, they located, identified, ﻿and visited as early as possible those children whose growth was not progressing appropriately in order to provide their caretakers with additional nutritional counseling. We refer to this process as routine growth monitoring*.*

The Level-1 and Level-2 Promoters weighed and measured the height of every under-2 child during a home visit when the child turned 3, 6, 12, 18, and 24 months of age. The visits also included nutrition counseling, vitamin A supplementation, and deworming with an oral medication (albendazole) for the child according to the schedule prescribed by the Ministry of Public Health and Social Welfare (*Ministerio de Salud Pública y Asistencia Social,* hereafter referred to as MSPAS).

In addition, we executed anthropometric censuses/*barridos* through which we weighed and measured every under-2 child. This is to be distinguished from the household censuses and mapping that took place at the initiation of Project activities as part of the CBIO process.

A small-quantity (20 g) of lipid-based nutrient supplement (Nutributter®) was also provided to beneficiary children in Area A for 4 months during 2013 as part of a partnership with a Guatemalan non-governmental organization, *Wuku’ Kawoq*, that donated this nutrient-dense food supplement. Nutributter® is a ready-to-use nutritional supplement that provides all vitamins and minerals required for the healthy growth of infants 6 to 12 months of age. The paste is packaged in 20 g sachets. The supplement was provided between May and August 2013 to all mothers of 6-18-month-old children who attended Self-Help Group meetings (described further below). This was very well received and encouraged attendance at the Self-Help Groups during the critical early stage of the Project.

### Peer-to-peer nutrition education through Care Groups

Care Groups enabled the Project to bring nutritional skills and knowledge to every mother with an under-2 child. In each village, the Level-2 Promoters trained a Level-1 Promoter who in turn trained a cadre of 5-12 volunteer mother peer educators known as *Comunicadoras* (referred to here as Care Group Volunteers). Each Care Group Volunteer was assigned 10–15 mothers of under-2 children in her community, and collectively they covered 100% of the target population. The Care Group Volunteers performed monthly home visits to each home in their catchment area and twice per month convened a group meeting for all mothers in their catchment area.

These group meetings were called Self-Help Groups (*Grupos de Autocuidado*). Participatory lessons for non-literate adult audiences were shared that emphasized the importance of exclusive breastfeeding during the first six months of life followed by the addition of complementary feeding at six months of age. The complementary feeding lessons promoted a diverse diet of locally available and affordable foods rich in protein, iron, and vitamins and with sufficient caloric content. Nutrition-related behavior change communication (BCC) lessons also emphasized water, sanitation, and handwashing (WASH) practices (point-of-use water purification and storage, proper feces disposal, and handwashing at critical moments) and promoted the need for vitamin A supplementation for children and consumption of vitamin-A-rich foods. Care Group Volunteers monitored uptake of these behaviors and reported their observations to the Level-1 Promoter. These reports were included in the Project’s monitoring and evaluation (M&E) data.

### Special support for undernourished children

Mothers of stunted and underweight children were targeted for either support groups for breastfeeding women (*Círculos de Madres Lactantes*) if their child was younger than 6 months of age or Positive Deviance (PD)/Hearth workshops (*Talleres Hogareños*) to learn proper complementary feeding practices if their child was 6- < 24 months of age (described below). These children were closely monitored. Cases of wasting were referred to the MSPAS health posts or clinics for the provision of nutritional supplementation and medical attention.

Locally available and affordable nutritious foods were identified utilizing the PD/Hearth methodology [23]. As part of the PD/Hearth methodology, a household survey of 288 mothers of under-2 children randomly selected using stratified cluster sampling was conducted in September 2012 in 30 Area A communities and established the baseline prevalence of stunting, underweight, and wasting (see Methods section below). Because Project implementation had not yet started in Area B, these same data were not collected there until 2013. The September 2012 survey data were utilized to identify “positive deviants” – the children in the same community who were at or above the normal weight and height for their age.

The mothers of these children were then interviewed to discover what and how they fed their children. The interviews revealed that these mothers were feeding their children locally available and affordable foods such as garden vegetables, wild greens, legumes, vegetable oil, fruits, and eggs. The Level-2 Promoters then designed a two-week menu cycle built around the traditional maize/tortilla dietary base but strongly supplemented with additional nutritious foodstuffs such as the ones identified above through the interview process.﻿ Assisted by Level-2 Promoters, to implement cooking lessons held in the kitchens of Level-1 Promoters or Care Group Volunteers, where the mothers in the Self-Help Groups received hands-on instructions and practice in preparing the foods of the next two-week menu cycle.

Table 1 summarizes the inputs, activities, and outputs of the Project’s nutrition interventions.

**Table 1:** Inputs, activities, and outputs of the Project’s nutrition interventions

Inputs	Activities	Outputs
Manual for Care Groups Manual for PD/Hearth intervention Community registers and maps Personnel: 26 Level-2 Promoters, 3 Care Group Supervisors, 3 Municipal Coordinators, 184 Level-1 Promoters, 779 Care Group Volunteers, 3 PEC Ambulatory Nurses, 5 PEC Auxiliary Nurses, 17 PEC Level-2 Promoters Scales for weighing children Measuring boards for determining height	Training of Level-2 and Level-1 Promoters and Care Group Volunteers in nutrition Establishment of Care Groups and Self-Help Groups Training of staff in Positive Deviance intervention and anthropometry Care Group meetings Self-Help Group meetings Growth monitoring of children 0- < 24 months of age PD/Hearth: weighing/measuring, survey of positive deviants, and design of menu and workshops *Talleres Hogareños* (community workshops on complementary feeding) for mothers of children 6- < 24 months of age with growth faltering Provision of anti-helminthic medication	28 Level-2 Promoters, 184 Level-1 Promoters, and 779 Care Group Volunteers trained in EBF, complementary feeding, anthropometry, and PD/Hearth intervention At least 93% of children 0- < 24 months evaluated for stunting, underweight, and acute malnutrition during each anthropometric census/*barrido* 31 children referred to a government health facility for management of wasting 468 children enrolled in the PD/Hearth program, which held 736 complementary feeding workshop sessions (*Talleres Hogareños*) 8,080 mothers educated in EBF and IBF and in proper complementary feeding practices

*EBF* Exclusive breastfeeding, *IBF* Immediate breastfeeding (after birth), *PD* Positive deviance, *PEC Programa de Extensión de Cobertura* (Extension of Coverage Program)

### Research questions

In this paper, we ask whether there were improvements in childhood nutritional status in the Project Area, whether these improvements were greater than those in comparison areas, and whether or not a dose–response effect was present in terms of a greater improvement in the part of the in Project Area A (with a longer duration of interventions) compared to Project Area B (with a shorter duration.

## Methods

As mentioned above, the Project was implemented for a longer duration in Project Area A (March 2012 through May 2015) than Project Area B (October 2013 through May 2015). We confirmed the comparability of Areas A and B based on analysis of the results of data collected from the baseline household survey carried out in January 2012. As described further in Paper 3 [3], there were very few statistically significant differences in socio-demographic characteristics of the households.

Using a quasi-experimental timeline series design with several non-randomized comparison areas, we obtained anthropometric data for under-2 children as follows: (1) In September 2012, the Project collected baseline height and weight measurements from 288 children in Area A using standard stratified cluster-sampling methodology. After the elimination of outliers, the data for 275 children were used to guide implementation of the PD/Hearth intervention, described above. (2) In June 2015, 300 children from Area A communities and 300 children from Area B communities using the same stratified cluster-sampling methodology, received height and weight measurements at the time of the endline KPC survey. Further details are available in Paper 2 in this series [2].

For each survey data set, the data were first analyzed with Epi Info 7 using z-scores to detect and eliminate outliers as specified by the World Health Organization reference tables for underweight (weight-for-age, abbreviated as WFA), stunting (height-for-age, abbreviated as HFA), and wasting (weight-for-height, abbreviated as WFH) [24]. Outliers were defined as measurements that were less than or greater than﻿ 6 standard deviations (SDs) for underweight and stunting (WFA and HFA) and less than or greater than 5 SDs for wasting (WFH). The September 2012 survey identified 13 outliers, and the June 2015 survey identified 12 outliers. With the outliers removed, the data sets were exported into Microsoft Excel, and﻿ undernutrition rates were calculated. These results were corroborated by two separate independent collaborators.

Endline KPC Survey anthropometric results for stunting, wasting, and underweight for the children from the Area A communities were compared with the Baseline KPC Survey results. Endline KPC Survey results for the children from Area A communities were compared with the Endline KPC Survey results for the children from Area B communities for underweight, stunting, and wasting﻿. Using Fisher’s midpoint test *p*-values were calculated for all of these comparisons utilizing WinPepi [25].

In addition to these three household surveys, we carried out anthropometric censuses/*barridos* (described above)*.* Beginning in June 2013 in the Area A communities, every under-2 child underwent weight and height measurement: in June and September 2013 and in January, August, and November 2014. In Area B, only two of these anthropometric censuses/*barridos* were conducted (in August﻿ and November 2014). They consumed a great deal of Project material and human resources, some of which was provided by the MSPAS Extension of Coverage (PEC) Program – especially the personnel required to conduct them. The loss of support from the PEC Program (described below) led to the premature termination of this activity.

During the anthropometric censuses, Level-2 Promoters, assisted by Level-1 Promoters, weighed and measured every under-2 child in their assigned communities during a home visit and utilized the WHO reference tables to identify all children < -2 SDs for stunting, wasting, and underweight. Curamericas Guatemala M&E staff then aggregated the data of the Level-2 Promoters into Microsoft Excel spreadsheets, aggregated by Area and by municipality, and reviewed every record, verifying and correcting, if necessary, the anthropometric classifications. Then, the prevalence of stunting, underweight, and wasting were computed. For the children from the Area A communities, November 2014 anthropometric census/*barrido* results were compared with the June 2013 anthropometric census/*barrido* results for underweight, stunting, and wasting. For the children from the Area B communities, November 2014 anthropometric census/*barrido* results were compared with the August 2014 census results. Final November 2014 census results for the children from the Area A communities were compared with the November 2014 census/*barrido* results for the children from the Area B communities﻿. Using Upton’s “N-1” chi square test, *p*-values were calculated for all of these comparisons utilizing WinPepi.

We utilized anthropometric data for the Northwestern Department of Guatemala (which includes the Departments of Huehuetenango and Quiché) from the 1999 and 2015 national Demographic and Health Surveys (DHSs) [17, 26]. Since the 1999 and 2015 DHSs did not provide information on the nutritional status of under-2 children from the rural area of the Department of Huehuetenango, we obtained the raw data from the DHS Program [27] and computed these values. The results were similar to the values in the reports for under-2 children and under-5 children for these two geographic areas when both rural and urban areas were combined (data not shown).

## Results

### Changes in nutritional status

#### Trends in stunting

**Fig. 1:**
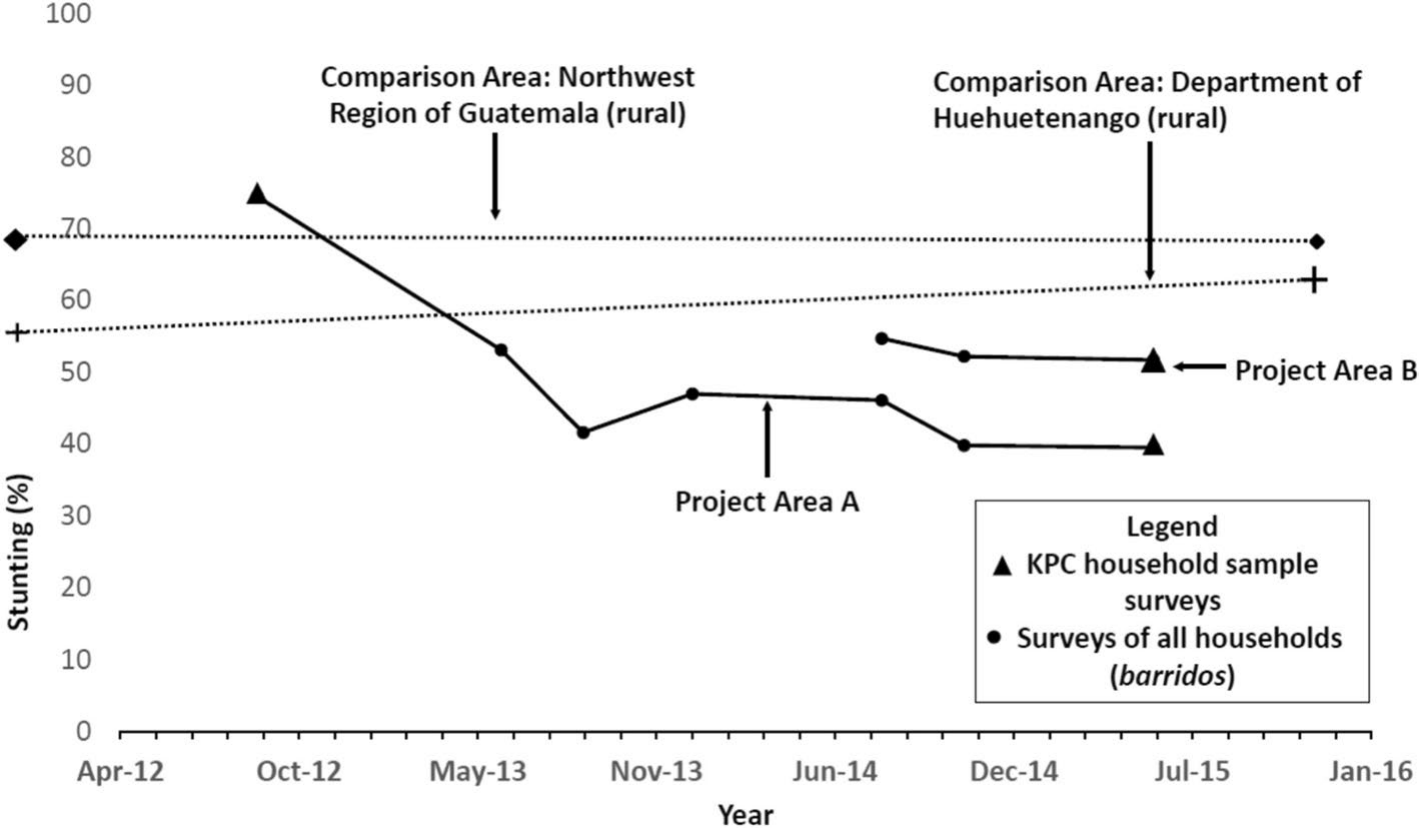
Changes in stunting of under-2 children in the Curamericas/Guatemala Project Area and under-2 children in comparison areas, 1999–2016. Note: The baseline values for the data from the comparison areas were collected in 1999

Figure 1 shows the changes in stunting for the children in the Project intervention areas compared to those for children in comparison areas. Tables 2, 3, 4, 5, and 6 contain the data used to create this figure. The prevalence of stunting (height for age) in Intervention Area A declined markedly from 74.5% at the time of the Baseline KPC Survey in September 2012 to 39.5% at the time of the Endline KPC Survey in June 2015. The anthropometric censuses/*barridos* obtained periodically in the interim confirm the early notable decline in stunting during the period. The statistical significance of the decline from baseline to endline is *p* < 0.001. In the Department of Huehuetenango (rural) and the Northwestern Region of Guatemala (rural), the prevalence of stunting did not decline between 1999 and 2015, and the endline prevalences were higher than for Project Areas A and B. At endline (June 2015), the prevalence of stunting in Area A was 39.5% compared to 51.7% in Area B, a difference that is statistically significant (*p* < 0.004). These findings demonstrate a marked and highly statistically significant decline in stunting in Project Area A in contrast to no decline in the comparison areas. During the last year of the Project, the prevalences of stunting in Project Area B were lower than in the non-Project comparison areas but higher than in Project Area A.

**Table 2:** KPC household survey anthropometric data for under-2 children from Area A communities

Anthropometric indicator and data source	Numerator	Denominator	%	95% confidence interval	*p*-value
**Stunted under-2 children who are < -2SD below normal height for age per WHO reference population**					
September 2012 Household Survey	205	275	74.5%	69.0, 79.6%	**0.000**
June 2015 Endline KPC Survey	116	294	39.5%	33.8, 45.3%	
**Wasted: under-2 children who are < -2SD below normal weight for height per WHO reference population**					
September 2012 Household Survey	13	275	4.7%	2.5, 7.9%	0.385
June 2015 Endline KPC Survey	9	294	3.1%	1.4, 5.7%	
**Underweight: under-2 children who are < -2SD below normal weight for age per WHO reference population**					
September 2012 household survey	82	275	29.8%	24.5, 35.6%	**0.009**
June 2015 Endline KPC Survey	59	294	20.1%	15.6, 25.1%

**Table 3:** KPC survey anthropometric data for under-2 children from Area B communities

**Anthropometric indicator and data source**	**Numerator**	**Denominator**	**%**	**95% confidence interval**	***p*-value**
**Stunted: under-2 children who are < -2SD below normal height for age per WHO reference population**					
June 2015 Endline KPC Survey	152	294	51.7%	45.8, 57.5%	N/A
**Wasted: under-2 children who are < -2SD below normal weight for height per WHO reference population**					
June 2015 Endline KPC Survey	13	294	4.4%	2.4, 7.4%	N/A
**Underweight: under-2 children who are < -2SD below normal weight for age per WHO reference population**					
June 2015 Endline KPC Survey	59	294	20.1%	15.6, 25.1%	N/A

N/A: not applicable

**Table 4:** Underweight, stunting, and wasting in under-2 children from Area A communities compared to children from Area B communities at the time of the June 2015 Endline KPC Survey

**Area in which children were weighed and measured**	**Numerator**	**Denominator**	**%**	**95% confidence interval**	***p*-value**
**Stunting—children < 2 years old who are < -2SD below normal height for age per WHO reference population**					
Area A communities	116	294	39.5%	33.8, 45.3%	**0.004**
Area B communities	152	294	51.7%	45.8, 57.5%	
**Wasting—children < 2 years old who are < -2SD below normal weight for height per WHO reference population**					
Area A communities	9	294	3.1%	1.4, 5.7%	0.515
Area B communities	13	294	4.4%	2.4, 7.4%	
**Underweight—children < 2 years old who are < -2SD below normal weight for age per WHO reference population**					
Area A communities	59	294	20.1%	15.6, 25.1%	1.00
Area B communities	59	294	20.1%	15.6, 25.1%

**Table 5:** Nutritional status measures from comparison areas outside of the Project Area for under-2 children

**Type of nutritional indicator**	**Area in which children were weighed and measured**			
	**Department of Huehuetenango rural only, under-2 children only (DHS data)**		**Northwest Guatemala (Departments of Huehuetenango and Quiché) (DHS data)**	
	**1999** [26]	**2015** [17]	**1999** [26]	**2015** [17]
**Stunting**	57.1%	64.6%	69.2%	68.2%
**Wasting**	1.9%	1.1%	2.5%	0.3%
**Underweight**	23.8%	18.4%	33.4%	19.8%

References: [17, 26]

*Note*: *DHS* Demographic and Health Survey. The sample sizes for the data for the Department of Huehuetenango were 105 in 1999 and 223 in 2015, and for the Northwestern Region, they were 179 in 1999 and 479 in 2015

**Table 6:** Results of anthropometric censuses/*barridos* of under-2 children weighed and measured in Area A and Area B between 2013 and 2014

**Month/year of *barrido***	**Number of children weighed and measured**	**Number stunted ** **(< -2SD HFA)**	**% stunted**	**Number wasted ** **(< -2SD WFH)**	**% wasted**	**Number underweight ** **(< -2SD WFA)**	**% underweight**
**Area A communities**							
June 2013	2,093	1,112	53.1%	40	1.9%	486	23.2%
September 2013	2,093	871	41.6%	28	1.3%	328	15.7%
January 2014	2,197	1,032	47.0%	18	0.8%	331	15.1%
August 2014	2,401	1,106	46.1%	10	0.4%	320	13.3%
November 2014	2,194	874	39.8%	7	0.3%	239	10.9%
**Area B communities**							
August 2014	2,198	1,203	54.7%	24	1.1%	442	20.1%
November 2014	2,051	1,071	52.2%	17	0.8%	317	15.5%

If the baseline prevalence of stunting in Area B was similar to that for Area A, we would see a decline in stunting in Area B, though one that was not quite as marked as in Area A. This is not surprising since the length of the Project intervention in Area B was less than half that for Area A, and the anthropometric censuses/*barridos* did not begin in Area B until less than a year before the end of the Project (August 2014). The findings are consistent with a dose–response effect since the improvements in nutritional status are more pronounced in Area A, where the nutrition-related activities took place for a longer period, than in Area B.

We obtained the prevalence of stunting for under-2 children in the comparison areas of the rural Northwestern Region of Guatemala (which includes the Departments of Huehuetenango and Quiché) and for the Department of Huehuetenango itself. In the Northwestern Region, there was no change in the prevalence of stunting between 1999 and 2016. In the Department of Huehuetenango, the prevalence of stunting actually increased from 57.1% to 64.6%.

#### Trends in wasting

Changes in wasting for the children in the Project Areas and in the comparison areas are shown in Tables 2, 3, 4, 5, and 6. The﻿ prevalence of wasting in Area A as measured at the time of the KPC surveys declined from 4.7% to 3.1%, a difference that was not statistically significant. Similar to our findings for stunting, the prevalence of wasting in Area A at endline was lower than the prevalence of wasting in Area B at endline (3.1% vs. 4.4%), but this very small difference was not statistically significant. The prevalence of wasting in the comparison areas of the Northwestern Region of Guatemala and the Department of Huehuetenango show similarly low levels. Because the differences are so small, we have elected not to present them in a figure. There is some discordance related to the measurement of wasting in the Project Areas A and B between the household KPC surveys, which found a higher wasting prevalence, and the anthropometric censuses/*barridos,* which found a lower wasting prevalence. Because of the small percentages of children who were wasted, these differences are minimal. The anthropometric censuses/*barridos* (Table 6) revealed that the prevalence of wasting was slightly lower in Project Area A than in Project Area B. The difference in August 2014 (0.4% in Area A compared to 1.1% in Area B) is statistically significant (*p* = 0.008) as is the difference in November 2014 (0.3% in Area A compared to 0.8% in Area B, *p* = 0.027). However, the difference arising from the KPC Endline Surveys in June 2015 (3.1% in Area A compared to 4.4% in Area B﻿) as shown in Tables 4 is not statistically significant.

#### Trends in underweight

**Fig. 2:**
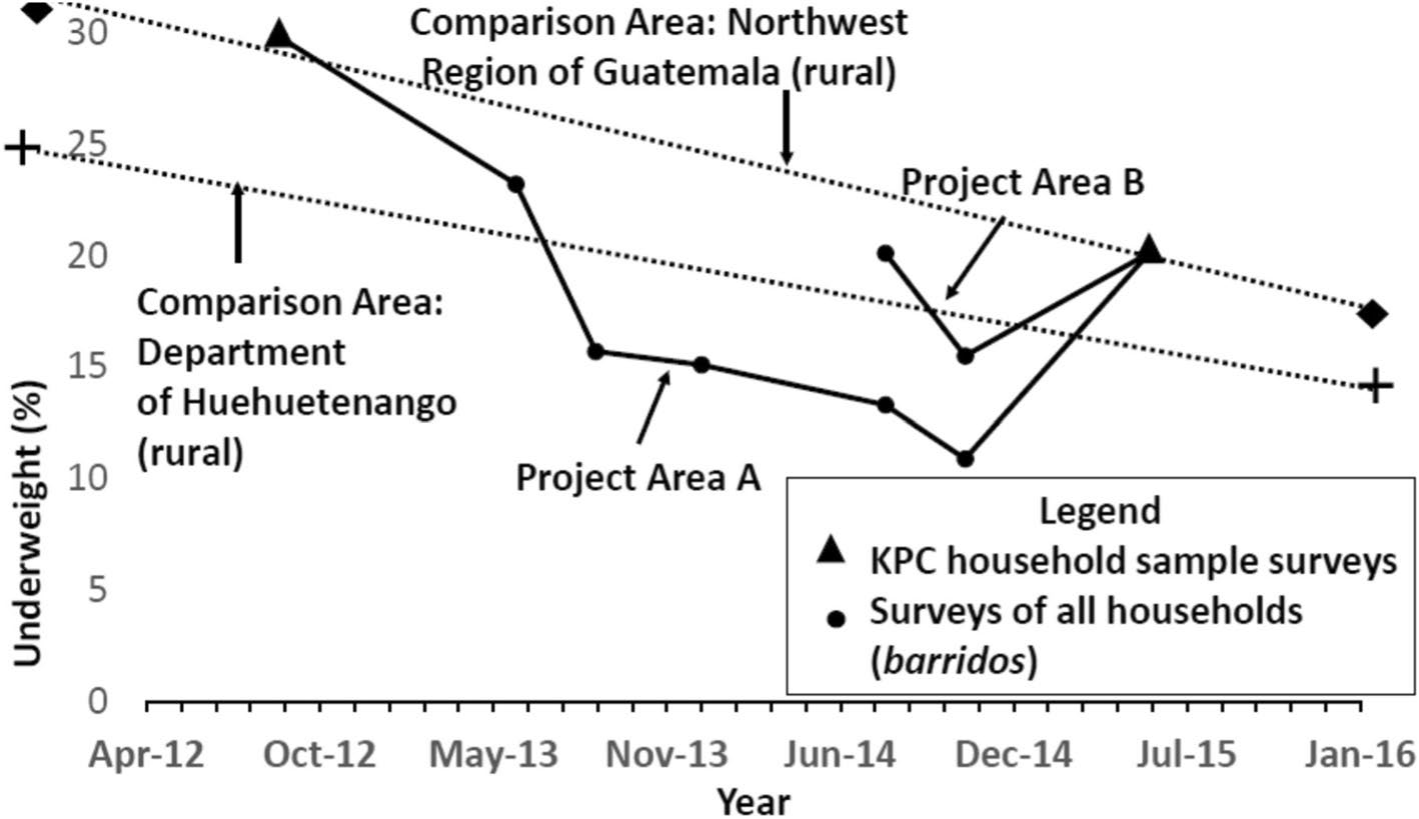
Changes in underweight for under-2 children in the Curamericas/Guatemala Project Area and under-2 children in comparison areas, 1999–2016. Note: The baseline values for the data from the comparison areas were collected in 1999

Figure 2 shows the changes in underweight, which reflect both stunting and wasting together, for the children in the Project Intervention Areas compared to those for children in comparison areas. Tables 2, 3, 4, 5, and 6 contain the data used to create this figure. Underweight declined significantly from baseline to endline in Project Area A (from 29.8% to 20.1%, *p* = 0.009). The prevalence of underweight in Project Area A and Project Area B at endline (June 2015) was the same (20.1%). The prevalence of underweight in the Northwestern Region of Guatemala declined from 27.6% in 1999 to 17.3% in 2015, while in the Department of Huehuetenango, the prevalence of underweight declined from 23.8% to 18.4% [17, 26].

Although the prevalence of wasting and underweight based on KPC data are somewhat higher than those based on the anthropometric census/*barridos*, the trends are similar in that they all show gradual improvements over time. Table 6, showing data for only the anthropometric censuses/*barridos,* demonstrates reductions in wasting and underweight in both Areas A and B as well as in stunting, as we have seen previously. Overall, these findings support the conclusion that the prevalence of underweight in Area A declined from baseline to endline and at a faster rate than in the comparison areas outside of the Project Areas (Table 5).

### Coverage of nutrition-related interventions

As discussed in paper 3 of this supplement [3], coverage of nutrition-related indicators improved significantly during the Project period. The practice of proper infant and young child feeding improved significantly in both Areas and was notable in Area A (from 53.0% to 74.3%, *p* < 0.001); the increase in Area B was less notable but still statistically significant (from 56.1% to 65.3%, *p* = 0.041). The prevalence of exclusive breastfeeding was high at baseline in both Areas A and B, and there was no significant change over time in Area A or B. There is some slight evidence of a dose–response effect for these nutrition-related output indicators as well. The difference-in-differences analysis presented in Paper 3 [3] indicates that outputs were more significantly favorable in Area A than in Area B for exclusive breastfeeding and infant and young child feeding. However, for many of the other indicators related to diarrhea prevention and treatment and WASH, improvements in Area B were equally pronounced as in Area A.

As shown in Table 7, ongoing growth monitoring every 3–4 months with the anthropometric censuses/*barridos* reached 93–100% of children enrolled in the Project census. In addition, growth monitoring occurred at the time of the regular home visits by the Level-2 and Level-1 Promoters, described previously. Nutritional counseling was provided at the time of the growth monitoring.

**Table 7:** Coverage of anthropometric censuses/*barridos* by Project Area

Date of data collection	Number of children 0- < 24 months of age who were weighed and measured		Total population of children 0- < 24 months of age (according to CBIO Community Registers)		Percentage of children 0- < 24 months of age who were weighed and measured	
	**Area A**	**Area B**	**Area A**	**Area B**	**Area A**	**Area B**
June 2013	2,093	0	2,093	N/A	100%	N/A
Sep 2013	2,093	0	2,093	N/A	100%	N/A
Jan 2014	2,197	0	2,197	N/A	100%	N/A
Aug 2014	2,401	2,198	2,548	2,215	94%	99%
Nov 2014	2,194	2,051	2,367	2,147	93%	96%

N/A: not applicable

As a result﻿ of growth monitoring, 468 children were identified with growth faltering and were enrolled in 52 PD/Hearth Workshops (with an average of nine children per workshop) and a total of 736 sessions. 17% of the enrolled children were no longer underweight at the end of the workshop.

There is considerable evidence that the population coverage of interventions for diarrhea prevention and treatment increased from baseline to endline in both Areas A and B, as described in Paper 3 in this supplement [3]; the safe storage of water, regular point-of-use water treatment, presence of an appropriate handwashing station, and appropriate handwashing practices all increased significantly in both Areas A and B, and safe disposal of child feces improved significantly in Area B. The use of oral rehydration therapy for the treatment of diarrhea increased significantly in Area A, and the percentage of mothers who reported that they gave more fluids to their children with diarrhea increased significantly in both Areas A and B. These changes likely contributed to the improvement in nutritional status observed by reducing the frequency or severity of childhood diarrhea. Unfortunately, there were no improvements in the indicator for same/increased food intake during the diarrheal episode.

## Discussion

The Curamericas/Guatemala Maternal and Child Health Project, 2011–2015, aimed to improve the nutritional status of under-2 children and devoted 30% of its effort toward this end. The goal was achieved in Project Area A where we observed significant improvements in stunting, wasting, and underweight. Most notably, stunting prevalence in Area A declined from 74.5% in September 2012 to 39.5% in June 2015, with five separate anthropometric censuses/*barridos* of﻿ all under-2 children during the interim confirming an overall downward trend in stunting.

No improvements in nutritional status were observed in Project Area B, where Project interventions were delivered over a considerably shorter period of time. Unfortunately, we do not have a baseline measure for stunting in Area B. If the baseline measure for Area B was similar to Area A’s, which seems plausible given the similar socio-demographic characteristics of the two Areas and their contiguous location, then an improvement in stunting would have been observed﻿ but not to the same extent as in Area A.

These findings lend support to our hypothesis that the nutritional improvements in Area A can be attributable to the Project and not to other extraneous factors since, in a limited way, Area B served as an imperfect comparison area given that the Project implementation there did not begin until more than halfway through the Project. Furthermore, the prevalence of stunting in Project Area A was similar to the comparison areas outside of the Project at baseline, and the prevalence of stunting in these comparison areas did not decline to the same degree as in Area A of the Project. These findings are relevant given the high prevalence of stunting in the Northwestern Region of Guatemala and the limited progress made in the reduction of stunting in the Northwest Region. The improvements in child nutrition in the Project area can be attributed to improvements in dietary practices (described further in Paper 3 [3]), improvements in childhood infection prevention and control (described further in﻿ Paper 3 [3]), and the empowering effects of the Care Group approach on mothers (described further in Papers﻿ 7 and 8 [7, 8]). Even though the promotion of exclusive breastfeeding was a prominent part of the nutrition program, there were no significant improvements in the level of this indicator in Area A or B, partly because the level of this indicator was already high at baseline (75.0% in Area A and 79.2% in Area B) [3]. Our findings here demonstrating improved nutrition-related practices and nutritional status through the use of multiple community-based interventions are consistent with those of other studies that have demonstrated the effectiveness of the Care Group Approach alone﻿ [28–30] and the PD/Hearth intervention alone [31].

We observed some discrepancies between the results of the anthropometric censuses/*barridos* and the Endline KPC Survey on the measures of underweight and wasting. Both of these measures can be relatively volatile, and thus the short length of time between November 2014, the date of the final anthropometric census/*barrido,* and June 2015, the date of the Endline KPC Survey, could possibly account for the discrepancy. By comparing data from Tables 2, 3, and 6, as shown in Table 8, we can see that wasting and underweight prevalence in the Project Areas increased significantly between November 2014 and June 2015. The percentage of children who were wasted increased from 0.3% to 3.1% in Area A and from 0.8% to 4.4% in Area B. The percentage of children who were underweight increased from 10.9% to 20.1% in Area A and from 15.5% to 20.1% in Area B during the same period. All of the increases in wasting and underweight are statistically significant. Possible explanations for these increases in wasting and underweight include general deterioration of the local health system and severe disruptions in the national Guatemalan health system. Corruption, mass resignations, and arrests of high government officials, among other factors, led to the closure of government health services in the Project Area after the failure to pay government health staff working in the Project Area for months. In addition, the *Programa de Extensión de Cobertura* (PEC/Extension of Coverage Program) of the MSPAS, which brought Ambulatory Nurses into the communities to provide critical preventive and treatment services such as immunizations and the treatment of sick children, abruptly ended in October 2014, leaving families without affordable and accessible health services for sick children. Consequences of the early termination of the PEC Program are explored further in Papers 3 and 5 of this supplement [3, 4].

**Table 8:** Comparisons of prevalence of wasting and underweight in Areas A and B between November 2014 and June 2015

**Project Area**	**Indicator**	**Survey date**		**Statistical significance**^a^
		**November 2014**	**June 2015**	
Area A	Wasting	0.3% (7/2,184)	3.1% (9/294)	*p* = 0.000
	Underweight	10.9% (239/2,194)	20.1% (59/294)	*p* = 0.000
Area B	Wasting	0.8% (17/2,051)	4.4% (13/294)	*p* = 0.000
	Underweight	15.5% (317/1,071)	20.1% (59/294)	*p* = 0.001

^a^Comparing result for November 2014 with June 2015

The effect of the termination of PEC is also reflected in the Endline KPC Survey, which shows significant declines in the coverage of key PEC-provided services such as child immunizations and supplementation of vitamin A, as described in Paper 3 of the supplement﻿ [3]. In addition, the Project’s vital events registration system showed a sharp rise in infant mortality during this period, as documented in Paper 5 [4].

Although measures of the prevalence of stunting are less volatile than those for underweight and wasting, the decline in the prevalence of stunting in Area A between September 2012 (74.5%) to June 2013 (53.1%) and from June 2013 (53.1%) to September 2013 (41.6%) are notable, particularly since there is a plateauing of the prevalence after that date. We do not have a good explanation for why this might be. There was a corresponding notable reduction in underweight during the same period in Area A, again followed by a plateauing of the level. We can only speculate as to possible reasons for this. Among them is the possibility that the Project was able to improve the nutritional status of a group of children whose caretakers were readily amenable to the efforts to improve their nutritional status while the remaining caretakers of undernourished children remained resistant to Project interventions. Another possible reason is the distribution of Nutributter® from May through August 2013 to children 6–18 month of age in Area A. A final explanation, which relates to the plateauing effect beginning in September 2013, is the deterioration of the PEC Program, discussed elsewhere, particularly since growth monitoring was an integral part of it.

The inclusion of the PD/Hearth intervention into the CBIO+ service platform must be noted. The CBIO’s use of community registers, maps, and routine home visitations facilitated the identification of both the positive deviants and the malnourished children needing follow-up attention. The Care Group training cascade provides a ready community infrastructure and volunteer labor force for the implementation of the PD/Hearth workshops (*talleres hogareños*). The Project’s PD/Hearth intervention also confirmed that there were locally available and affordable nutritious foods. Other programmatic interventions that could have contributed to the nutritional gains reported here include the periodic provision of micronutrient supplements and deworming medication.

The contribution of the Care Groups also must be noted and goes beyond the *talleres hogareños.* This is revealed by the statistically significant increases from the Baseline to the Endline KPC Surveys in both Project Areas regarding key household behaviors that impact nutrition that are presented in Paper 3 of this supplement [3]. These include proper Infant and Young Child Feeding IYCF) (i.e., the percentage of infant and young children 6- < 24 months of age fed according to a minimum of appropriate feeding practices); prompt care seeking and treatment of children with symptoms of possible pneumonia; use by child caretakers of oral rehydration therapy (ORT) during a diarrheal episode; appropriate point-of-use treatment and storage of water; and hand washing at critical moments such as after defecating, after cleaning a child who had defecated, before preparing food, and before feeding a child.

Even though the exclusive breastfeeding (EBF) indicator assessed in Paper 3 [3] showed no improvement in either Area A or Area B, this indicator was measured by asking the mother only about her feeding practices during the previous 24 h. It does not take into account other important qualitative features of optimal EBF that were stressed during all educational sessions that mothers received, namely: (1) the importance of immediate breastfeeding within the first hour of birth, (2) the technical aspects of proper breastfeeding (position and so forth), (3) giving the infant enough time to feed until completely satisfied, (4) offering the breast to the child frequently throughout the day and night, and (5) having the confidence that EBF is the best way to provide optimal nutrition to one’s infant. It is likely that these unmeasured aspects of EBF did improve during the course of the Project. The quality of breastfeeding might have been improved as a result of the Project’s promotion of EBF in the Self-Help Groups and the support groups for lactating mothers (*Círculos de Madres Lactantes*) provided by Care Group Volunteers*,* who were both monitoring and encouraging this behavior at the household level.

Data from the anthropometric censuses/*barridos* indicate that the percentage of under-2 children in Area A who were stunted and underweight declined gradually from 53.1% to 39.8% and from 23.2% to 10.9%, respectively, between June 2013 and November 2014, as shown in Table 6. This strongly suggests that the quality of feeding practices for these children improved and/or the frequency of serious illness in these children diminished, even though we do not have any data to confirm this.

Our findings support the thesis that the CBIO+ Approach’s combination of routine home visitations guided by community registers and maps; the Care Group training cascade of bringing skills, knowledge, and lifesaving behavior change to every home; and the PD/Hearth intervention for empowering communities to improve child feeding practices with their own available and affordable foods were together able to produce significant improvements in child nutrition.

### Limitations

There are three notable limitations of our study. The first is the lack of true baseline anthropometric data. The baseline KPC household survey carried out in January 2012, which included all population-based indicators for the research, included the measurement of weight but not height. This was an oversight on our part and we also later had reason to suspect that there were errors in the weight measurements that were obtained at that time. As a result, we decided to carry out a separate household survey in September 2012 at which time both height and weight were measured, with amplified training for the interviewers and enhanced supervision. These are the best baseline data we have even though they were collected 12 months after the Project officially began.

Another limitation is that the anthropometric censuses/*barridos*﻿ were conducted infrequently and irregularly, and there were only two of these carried out in Area B. This was a resource-intensive activity and required support from the PEC staff, which began to dwindle in early 2014 and then the PEC Program shut down entirely later that year. The anthropometric censuses/*barridos* were conducted regularly in Area A in 2013 (June, September, and January), but thereafter there were delays because of a lack of PEC personnel, so they were not performed again until August and November 2014. Area B was not programmatically ready until August 2014 and then received an additional regularly scheduled anthropometric census/*barrido* in November 2014. However, by then the loss of PEC support caused the termination of this activity in both Areas.

The third limitation is that the comparison data used from outside the Project area are for a small sub-sample of rural under-2 children from the Northwestern Region of Guatemala and from the Department of Huehuetenango. We have no assurance that these data are representative of the children in these areas since the sampling design was not constructed in this way. In the 1999 DHS, there were only 179 and 105 children in the sample for the Northwestern Region and for the Department of Huehuetenango, respectively, giving further caution to the interpretation of these data.

### Policy implications

The problem of childhood undernutrition and stunting, in particular, among the children in the Project Area and among Indigenous children in rural Guatemala more broadly is a serious, complex, multifactorial problem that has been difficult to resolve in spite of repeated efforts and investments of large sums of money [32]. Early classical studies from Guatemala beginning in 1969 assessed the benefits of food supplements for linear growth in small study populations [33, 34].

Olney and colleagues [35] reported the findings of a cluster-randomized, controlled intervention trial assessing the effectiveness of family food supplementation with monthly behavior change communication (BCC) sessions on health and nutrition and preventive health services on stunting during the first 24 months of life in the largely Indigenous population of the Department of Alta Verapaz in Guatemala. Receipt of the monthly ration was dependent upon attending a BCC session immediately before the food distribution along with a review of the family’s health cards, pre- and postnatal checkups when appropriate, and monthly growth monitoring and promotion. The prevalence of stunting at 24 months of age declined by 6.5–11.1 percentage points depending on the size of the monthly food ration and the type of food ration. Fortified foods are considered by the rural Indigenous population in the region to be expensive [36] and are not widely used nor have they, of course, resolved the problem of childhood stunting in rural indigenous children in Guatemala.

A recent systematic review of studies of randomized-controlled interventions to improve linear growth in children 6- < 24 months of age in low- and middle-income countries [37] found that micronutrient and food supplements are effective in accelerating linear growth and reducing the prevalence of stunting. However, food supplementation as an approach to improving childhood nutrition is fraught with numerous operational, political, and financial challenges.

The communities in our study receiving the greatest duration and intensity of nutrition-related interventions showed the greatest improvement in the prevalence of stunting. This trend preceded the distribution of small-quantity lipid-based nutrient supplements during a relatively brief period in programmatic terms: four months. The Care Group Approach has been used widely in many countries, mostly by non-governmental organizations, and it has achieved remarkable successes in the coverage of nutrition-related interventions and in undernutrition without food supplementation [38–40], though we are not aware of any reports of the effectiveness of the Care Group approach in reducing stunting.

The CBIO+ Approach used here is worthy of further investigation and replication. The government of Guatemala recently planned to begin a US$100 million program to address chronic undernutrition/ stunting (*Crecer Sano*: Guatemala Nutrition and Health Project) [41]. However, because of the COVID-19 pandemic, these funds were diverted to support the care of hospitalized patients with COVID-19 [42]. 

Our﻿ findings are consistent with those of Tschida et al. [43] who emphasize the importance of a broad and comprehensive approach to reducing child stunting in Guatemala. Our findings provide strong support that a comprehensive, integrated set of community-based inputs and activities that includes the Care Group approach, the PD/Hearth intervention, frequent growth monitoring, and workshops for mothers of 6- < 24-month-old children with growth faltering can be effective in improving the nutritional status of Indigenous children in rural Guatemala. Such an approach could be effective in other areas of the world with high levels of childhood undernutrition.

## Conclusion

Taken together, these findings support the hypothesis that the Curamericas/Guatemala Maternal and Child Health Project, 2011–2015, was able to improve the nutritional status of under-2 children in an area with a high prevalence of childhood stunting. A census-based approach was used to reach all households with children younger than 2 years of age; promote healthy household behaviors and nutritional practices through peer-to-peer counseling and periodic growth monitoring of all children; provide vitamin A, anti-helminthic medication, and small quantity lipid-based nutrient supplements; and hold Positive Deviance/Hearth workshops to assist the mothers of undernourished children in improving household and nutritional practices.

## Disclosure statement

No potential conflict of interest was reported by the authors.

## Data Availability

All of the Project reports, de-identified data, as well as publications about the Expanded CBIO+ Approach cited in this article are available from the corresponding author on request.

## Abbreviations

BCC: Behavior change communication
CBIO: Census-Based, Impact-Oriented Approach
DHS: Demographic and health survey
HFA: Height-for-age
KPC: Knowledge, practice, and coverage (household survey)
M&E: Monitoring and evaluation
MSPAS: *Ministerio de Salud Pública y Asistencia Social* (Ministry of Health and Social Assistance)
PEC: *Programa de Extensión de Cobertura* (Extension of Coverage Program)
PY: Project Year (October 1 through September 30)
PD: Positive deviance
SD: Standard deviation
URC: University Research Corporation
WASH: Water, sanitation and hygiene
WFA: Weight-for-age
WFH: Weight for height
WHIP: Western Highlands Integrated Project
WHO: World Health Organization

## Footnotes

1 For under-2 children, length (measured with the child supine) is routinely measured while for children 2 years of age and older, height (measured with the child standing) is routinely measured. We use the term height throughout this article even though for under-2 children length was actually measured.
